# Immobilization of a *Pleurotus ostreatus* Laccase Mixture on Perlite and Its Application to Dye Decolourisation

**DOI:** 10.1155/2014/308613

**Published:** 2014-05-08

**Authors:** Cinzia Pezzella, Maria Elena Russo, Antonio Marzocchella, Piero Salatino, Giovanni Sannia

**Affiliations:** ^1^Dipartimento di Scienze Chimiche, Università di Napoli Federico II, via Cynthia 4, 80126 Napoli, Italy; ^2^Istituto di Ricerche sulla Combustione, Consiglio Nazionale delle Ricerche, Piazzale V. Tecchio 80, 80125 Napoli, Italy; ^3^Dipartimento di Ingegneria Chimica, dei Materiali e della Produzione Industriale, Università degli Studi di Napoli Federico II, Piazzale V. Tecchio 80, 80125 Napoli, Italy

## Abstract

In the present study, a crude laccase preparation from *Pleurotus ostreatus* was successfully immobilized on perlite, a cheap porous silica material, and tested for Remazol Brilliant Blue R (RBBR) decolourisation in a fluidized bed recycle reactor. Results showed that RBBR decolourisation is mainly due to enzyme action despite the occurrence of dye adsorption-related enzyme inhibition. Fine tuning of immobilization conditions allowed balancing the immobilization yield and the resulting rate of decolourisation, with the adsorption capacity of the solid biocatalyst. In the continuous lab scale reactor, a maximum conversion degree of 56.1% was achieved at reactor space-time of 4.2 h. Stability and catalytic parameters of the immobilized laccases were also assessed in comparison with the soluble counterparts, revealing an increase in stability, despite a reduction of the catalytic performances. Both effects are most likely ascribable to the occurrence of multipoint attachment phenomena.

## 1. Introduction

The ever-increasing attention towards the design of industrial processes with low environmental impact and high sustainability has encouraged the search for new biocatalysts to be employed as green tools for several industrial applications. In this “greener” perspective, laccases (*p*-diphenol-dioxygen oxidoreductases; EC 1.10.3.2) represent an interesting class of biocatalyst, being able to oxidize a wide spectrum of aromatic compounds along with reducing molecular oxygen to water [1]. Besides the exploitation in several applicative fields, such as in food sector, paper and pulp industry, biosensing, polymer functionalization, and textile industries [2], laccases have attracted growing interest for their successful use in bioremediation processes [3], particularly for the treatment of dye-containing wastewaters [4, 5], thanks to the structural similarity of many textile dyes with laccase natural substrates. In particular, laccases from the white rot fungus* Pleurotus ostreatus* have been extensively studied for their decolourisation ability towards several classes of dyes and wastewater models [6–8]. Reported data indicated that the extent of decolourisation depends on the laccase isoform used [6] and on the chemical class of the dye [8].

Even though laccase efficacy in dye conversion has been extensively demonstrated [9], there are still many constraints to their application for real wastewater treatment, due to the hard conditions characterizing real wastewaters (extreme pH values, very high ionic strength, presence of surfactants, cheating agents, etc.) and the huge volume of polluted waters demanding remediation. Both constraints clearly highlight the need for the choice of very robust catalyst for the process. Enzyme immobilization has widened the scope of laccase application allowing not only reusing of the biocatalyst, with a benefit in terms of costs, but especially improving enzyme performances, leading to higher activity and stability at extreme pHs, elevated temperatures, or in organic solvents and improved thermostability. Such enhanced features have encouraged the use of immobilized laccases in several applications, as recently reviewed by Fernández-Fernández [10]. Different methodologies have been reported for laccase immobilization, such as adsorption, entrapment, encapsulation, covalent binding, and self-immobilization. The choice of the most suited method clearly depends on application that laccase is devoted to. Particularly, covalent binding is the most widely applied method for exploitation of immobilized laccases in industrial applications [11–13].

Covalent immobilization represents an attractive option to obtain enzymatic catalyst for wastewater treatment. This technique provides different advantages: (i) it prevents enzyme leakage even under harsh conditions; (ii) it facilitates enzyme use in continuous, packed bed, stirred tank, and fluidized bed reactors; (iii) it causes stabilization of the enzyme tertiary structure, usually as a consequence of multipoint attachment of the enzyme to the support, providing enzyme rigidity. The stabilization provided by covalent bonding is usually counterbalanced by partial enzyme deactivation. This negative effect can be mitigated by carefully optimizing the immobilization conditions in order to maximize the ratio between immobilized enzyme activity and activity of the primary enzyme solution.

Decolourisation of several dyes has been achieved by means of laccase covalently bound to different supports, such as alumina oxide pellets [14], controlled porosity-carrier beads [15], and epoxy-activated supports [13]. Effective dye removal was also carried out by* C. unicolor* laccase covalently immobilized on mesostructured siliceous cellular foams (MCGs) [16] or on supports functionalized with epoxy groups [2]. In the latter case, the degradation of different classes of dyes was reported, along with an improvement of stability toward pH, temperature, and storage of the immobilized enzyme. Entrapment of laccase has also been used as an immobilization approach for environmental applications. Chitosan-coated alginate beads have been packed in a fixed bed reactor and employed in RBBR decolourisation, achieving up to 70% decolourisation [6]. Alginate beads differently modified with chitosan or polyethylene glycol (PEG) have also shown to effectively decolourise several different dyes [17–19]. Entrapment in hydrogel structures has also been applied, for example, in the immobilization of a* T. versicolor* laccase, resulting in low substrate affinity but improvement in storage stability and in the decolourisation of Acid Orange 52 [20].

An important aspect to be considered in the design of an ideal immobilized biocatalyst is the cost related to the support and to the enzyme to be linked to it. In this study, a low-cost laccase mixture was obtained from* P. ostreatus *culture broth after optimization of laccase production conditions and one-step protein enrichment, as described by Palmieri et al. [6]. On the other hand, the search for a cheap solid support has oriented our study toward the choice of a siliceous material, perlite. Perlite is an amorphous aluminium silicate with more than 70% content of silica. Besides exhibiting the advantages of an inorganic carrier with respect to organic ones, such as a greater mechanical stability and resistance toward microbial attack and organic solvents, perlite turns out to be a cheaper alternative in comparison with other reported inorganic supports such as silica gels, alumina, and zeolites [21]. The choice of such an inert support implies that its surface has to be properly modified in order to offer functional groups for protein binding. Surface modification of these materials can be easily achieved and their reactivity may be finely tuned in the derivatization steps [22].

In this work, covalent immobilization of laccase on activated siliceous support, perlite, was investigated and the immobilized biosystem was tested for its potential exploitation in dye decolourisation using the reactive dye RBBR as model substrate. The immobilization process was properly optimized with reference to the immobilization yield and to the dye adsorption capacity of the solid biocatalyst. Stability and catalytic parameters of immobilized laccases were also assessed in comparison with the soluble counterpart.

## 2. Materials and Methods

### 2.1. Fungal Culture and Crude Laccase Extraction

*Pleurotus ostreatus* (type: Florida ATCC number MYA-2306) was maintained through periodic transfer every 3 weeks, at 4° on agar plates containing 24 g/L potato dextrose and 5 g/L yeast extract. Medium components were supplied by Difco Laboratories (Detroit, MI). Mycelium growth and crude laccase mixture extraction were carried out following the procedures described by Faraco et al. [7].

The crude laccase mixture extract is characterized by four isoforms (POXA1b, POXA3a, POXA3b, and POXC). The activity of the POXC isoform accounts for about 99% of the total mixture activity [7]. The laccase mixture used in the immobilization trials displays a specific activity of 70 U/mg.

### 2.2. Dye

The anthraquinonic dye Remazol Brilliant Blue R (RBBR) was purchased from Sigma-Aldrich. Powder purity was 50%. Dye concentration was measured by recording optical absorbance at 592 nm. The extinction coefficient (*ε*_592_ = 9,000 M^−1^ cm^−1^), referred to total powder concentration, was corrected taking into account the purity. All other reagents were purchased from Sigma-Aldrich with a ≥98% purity.

*Laccase Activity Assay. *Laccase activity was assayed at 25°C by monitoring the oxidation of ABTS at 420 nm (*ε*_420_ = 36 × 10^3^ M^−1^ cm^−1^) [6]. The assay mixture contained 2 mM ABTS in 0.1 M sodium citrate buffer (pH 3.0).

*K*_*M*_ values were estimated using the software GraphPad Prism (GraphPad Software, La Jolla, CA, USA; http://www.graphpad.com/) on a wide range of substrate concentrations (0.05–3 mM). Enzyme activity was expressed in international units (IU).

### 2.3. Protein Determination and Electrophoresis

Protein concentration was determined using the BioRad Protein Assay (Bio-Rad Laboratories, Segrate (MI), Italy), with bovine Serum albumin (BSA) as standard.

### 2.4. Perlite Pretreatment and Derivatization

Perlite (SIPERNAT 22©) was supplied by Degussa (Hanau, Germany). Solids (density about 1,026 kg/m^3^) were sieved in the range of 90–150 *μ*m. Perlite was pretreated with 1.2 M HNO_3_ at 60°C for 4 hours and then extensively washed with water and dried at 60°C. Solids were vigorously fluidized with water to remove fines. The minimum fluidization velocity estimated according to Fan [23] for the liquid-solids system is about 4·10^−6^ m/s.

Silanization is a crucial step with regard to subsequent reproducibility of the chemical functionalization. However, the surface coverage, orientation, and organization of reactive groups are still a subject of controversy [24]. Despite resulting in a lower surface concentration of amino groups in comparison with reaction in organic solvents, aqueous silanization with APTS (aminopropyltrimethoxysilane) has been selected for this study, since it was demonstrated to result in a more stable and uniform immobilized enzyme layer [25].

Glutaraldehyde is a bifunctional molecule which has been extensively used as an enzyme immobilizing agent. Although there are many discussions on the composition of the glutaraldehyde solution (monomeric and polymeric forms) and on the structures responsible for its properties, it is generally accepted that it is capable of reacting with surface amine groups of enzyme and carriers, through the formation of Schiff's bases and Michael's adducts [26].

Carrier activation was carried out as follows: (i) 0.2 g of dry pretreated perlite was mixed with APTS at a concentration from 0.4% to 4% in 5 mL distilled water and incubated at 80°C for 2 h under constant mixing; (ii) the suspension was washed thoroughly with 50 mM sodium phosphate (NaP) buffer, pH 6.5, and treated with glutaraldehyde solutions and dissolved at different concentrations, in the same buffer, for 2 h at room temperature; (iii) the activated perlite was extensively washed with the overcited buffer and finally incubated for 1 h with 5 mL of a solution of laccase mixture (22 U/mL) in 50 mM NaP buffer, pH 6.5, at room temperature. Residual active glutaraldehyde was inactivated by 1 h incubation with 100 mM glycine at room temperature.

### 2.5. Adsorption Experiments

Dye adsorption on unreacted particles was determined by incubating 0.2 g of particles in a RBBR solution at preset concentrations. All the experiments were performed in the conditions usually adopted during dye conversion tests, 20 mM sodium acetate (NaA) pH 4.5 at room temperature. Dye concentration in the liquid phase was measured as optical absorbance at 592 nm. The dye adsorption was highlighted by the decrease of the optical absorbance in the liquid phase as well as by an increase of particles colouration. The procedure adopted for each experiment was (i) dispersion of particles in dye solution; (ii) as the dye concentration in the liquid did not changed anymore (achievement of equilibrium between solids and liquid), the liquid was replaced with fresh dye solution. The cyclic operation was repeated until no change in the initial concentration of dye was observed. The overall amount of adsorbed dye during each cycle was calculated from the mass balance referred to the dye.

### 2.6. Assay of Immobilized Enzyme Activity

The activity of laccase immobilized on perlite was estimated by measuring the oxidation rate of 2,2′-azino-bis (3-ethylbenzothiazoline-6-sulphonic acid) (ABTS) in a recirculating fixed bed reactor previously designed for the assessment of activity of enzymes immobilized on granular solids [13]. The device was equipped with a fixed bed reactor, loaded with biocatalyst particles. It was operated by circulating the liquid phase containing the substrate between the fixed bed and a mixed tank according to the procedure reported by Russo et al. [13]. The increase of optical absorbance at 420 nm was assessed by online measurements on the circulating liquid phase in the mixed tank. The operating conditions were purposely selected in order to prevent mass transfer limitations during the enzymatic conversion: the assay was carried out under kinetic controlled regime. The operating conditions were total reaction volume (tank, tubes, fittings, and inert fixed bed) 72 mL, tubular reactor packed with 0.18 mL solid biocatalyst and liquid circulation rate at volumetric flow rate 20 mL/min.

### 2.7. Fluidized Bed Reactor for RBBR Conversion

**Figure 1:**
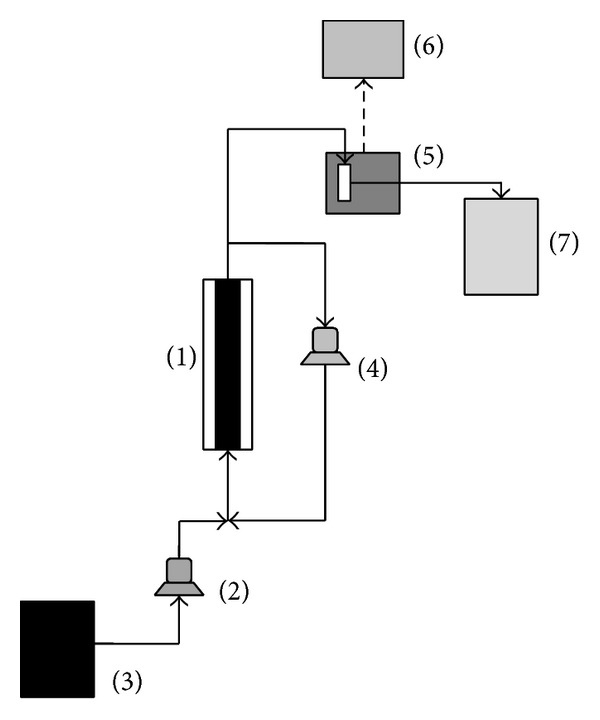
Apparatus equipped with fluidized bed reactor adopted for RBBR conversion by means of immobilized laccases. (1) Fluidized bed reactor; (2) peristaltic pump; (3) feed tank; (4) recirculation gear pump; (5) flow-cell and spectrophotometer; (6) data acquisition unit; (7) waste tank.

Conversion of RBBR by immobilized laccases was investigated in a fluidized bed reactor continuously operated with respect to the liquid phase. A sketch of the apparatus is shown in Figure 1. Biocatalyst particles were loaded in a cylindrical vessel (2.5 cm ID, 30 cm long). Solid particles were fluidized with the liquid stream delivered by a gear pump (VG 1000 digit, Verder). The dye-bearing liquid solution was fed at the reactor by means of a peristaltic pump. The liquid was recirculated through the reactor at volumetric flow rate (*Q*_*r*_) by means of a peristaltic pump (Miniplus, Gilson). Dye concentration in the outlet stream was measured continuously by means of a spectrophotometer (Cary 50, Varian Inc.) equipped with a flow-cell. Optical absorbance was measured at 592 nm. Dye conversion was carried out in conditions assessed as optimal for RBBR decolourisation, 20 mM sodium acetate buffer pH 4.5 at room temperature [6]. The minimum liquid flow rate required for the solid fluidization, estimated assuming the crude solid density, for example, without any adsorbed species, was about 0.10 mL/min.

Total liquid volume was set at 71 mL. The test procedure was as follows:

- saturation of the catalyst with the dye. The reactor was fed with dye bearing liquid stream at 12.4 mL/min (more than 100 times the expected minimum fluidization rate) without stream recycling. The liquid flow rate was enough to fluidized particles. The reactor space-time (*τ*≅4 min⁡) was set to saturate the solids with dye at a negligible enzymatic conversion; solid saturation was accomplished within 2 hours;
- as the solid was saturated, the feeding flow rate *Q* was decreased to the set value and the liquid was recycled (*Q*_*r*_ = 13 mL/min). A series of steady state regimes was investigated by setting *Q* in the interval 0.3–13 mL/min. The reactor was operated at the preset *Q* until dye concentration in the reactor approached a steady value. The dye conversion was assessed under steady state conditions.

## 3. Results and Discussion

### 3.1. Optimization of Immobilization Process on Activated Perlite

Functionalization of perlite surface has been achieved by two main steps: (i) silanization with a trifunctional organosilane agent APTS that provides the reactive amino groups susceptible to the following activation; (ii) reaction with a crosslinking reagent, glutaraldehyde.

In this section, results on the optimization of immobilization of crude laccase preparation on perlite are reported.

Activity of laccase immobilized on perlite was measured adopting a specifically designed device as described in Section 2. Immobilization yield (*Y*) is the objective parameter assessed to optimize the immobilization protocol. *Y* was defined as the ratio between laccase activity expressed by the solid biocatalyst and total activity in the liquid solution at the beginning of the immobilization processes.

Process optimization has been carried out assessing the effect of the following operating conditions on the immobilization yield: glutaraldehyde concentration, pH and buffer composition of immobilization solution, time and temperature of incubation, total activity, and total protein contents.  Table 1 reports the results in terms of immobilized activity and yield for each run. The operating conditions of each run are also reported.

#### 3.1.1. Effect of Glutaraldehyde Concentration

The effect of the results reported in Table 1 indicates that, at 1% and 2.5% glutaraldehyde concentrations, immobilization yield is higher when incubation is performed at RT for shorter time (R1, R3), with respect to overnight incubation at 4°C (R2, R4). No laccase activity or proteins were detected in the recovered supernatant at the end of incubation, indicating that 100% of the initial protein content was bounded to the carrier. The lower immobilization yield found in R2 and R4 could be ascribed to further interaction of enzymes with glutaraldehyde molecules for prolonged incubation time, causing laccase inactivation. When glutaraldehyde concentration is raised up to 5%, immobilization yields are almost comparable at RT for 4 h or overnight at 4°C (see R5 and R6). It is conceivable that, at higher concentration, glutaraldehyde-glutaraldehyde interactions prevail on glutaraldehyde-protein ones, thus reducing the negative effect on the enzyme observable at longer incubation times. Taken together, these results indicate that the reactive groups, made available by using glutaraldehyde concentrations in the range 1% to 5%, are sufficient to bind to all proteins present in the crude mixture, resulting in a maximum immobilization yield of about 34%. When immobilization experiments were carried out further lowering glutaraldehyde concentration down to 0.5%, a comparable yield (32%) was achieved (see R10). Thus, glutaraldehyde concentration was set at 0.5% for further immobilization experiments.

#### 3.1.2. Effect of pH and Buffer Composition

One of the factors that can affect enzyme immobilization is the pH value of the coupling mixture. The optimal pH value should be the compromise between conditions favoring enzyme stability and those promoting the nucleophilic attack of protein reactive groups to the glutaraldehyde functionalized support. The results obtained performing laccase immobilization at pH values 5.5, 6.5, and 7.5 are listed in Table 1 (R17–R22). The immobilization yield was almost constant in the neutral pH range (~50%) and decreased at 19% at low pH. Except for test carried out at low pH, no laccase activity or proteins were found in the recovered supernatant at high pH (6.5 and 7.5). At low pH, about 25% of the initial laccase activity was detected in the supernatant.

As reported in Table 1, immobilization at pH 5.5 was carried out in a buffer containing a different counterion, sodium citrate instead of sodium phosphate buffer at the same molarity. To complete the scenario of the combined pH buffer effects, tests were also performed at the three investigated pH values adopting citrate-based buffer, the McIlvaine buffer. Under these conditions (R20 through R22), a significant reduction of immobilization yield was observed at all the tested pH, with up to 28% of the initial laccase activity recovered in the supernatants.

These findings could be a consequence of the heterofunctional nature of the activated matrix: after glutaraldehyde activation, the support may expose unreacted amino groups which confer some ionic exchanger features to the support [22]. In such heterofunctional matrices, a first ionic adsorption of the protein on the amino groups of the support was found to occur before the covalent reaction between glutaraldehyde activated sites and the enzymes occurs. A different counterion selectivity (citrate > phosphate) towards the unreacted amino groups of the support could explain the observed effect, by assuming that most of the positively charged groups on the support are shielded by citrate (whose concentration increases lowering pH in McIlvaine buffer composition). Such charge-shielding effect would hinder the first ionic interchange of the protein on the amino groups of the supports, thus impairing immobilization yield.

#### 3.1.3. Temperature and Time of Incubation

The effect of temperature on the immobilization yield was assessed comparing the runs R1–R10. The immobilization yield assessed for tests carried out at incubation temperature as low as 4°C did not changed with respect to that assessed at room temperature. In addition, extending the incubation time to overnight incubation (at 4°C) caused a decrease in immobilization yield (compare R1–R3 with R2–R4) or resulted in almost comparable yields (compare R5 to R6) with respect to 4 hours room-temperature incubation. When laccase activity and total protein content were determined in the liquid supernatant as a function of the incubation time, both measured values became negligible after 15 minutes incubation, indicating the immobilization of almost the entire protein content of the crude mixture occurred. Hence, further experiments were carried out at incubation time set at 1 h without temperature control.

#### 3.1.4. Effect of Total Activity and Total Protein Contents

**Figure 2:**
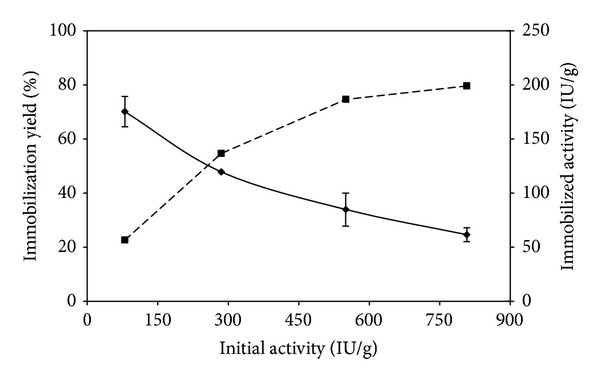
Immobilization yield (solid line) and immobilized activity (dashed line) versus laccase initial activity.

Some immobilization tests were carried out at the ratio between initial laccase activity and mass of dry support set in the range of 80–800 IU/g (experiments R11–R13). For a given amount of dry support, results showed that the immobilization yield decreased with the amount of initial laccase activity while the immobilized activity (IU/g) approached a constant value (Figure 2). This observation could be interpreted taking into account protein overcrowding on the carrier surface that may reduce substrate accessibility to the active site. However, results of the test series of tests R14 through R16 enabled us to rule out this hypothesis. As a matter of fact, tests carried out at a given activity (IU/g) and at protein content increased by adding BSA were characterized by absence of immobilization yield, as it would have been expected if the above interpretation was verified. Moreover, it is worth noting that proteins were completely bounded to the support even at the highest concentration investigated. Both series of data (R11 through 13 and R14 through R16) can be accounted for considering that access of substrate to immobilized enzyme was restricted by the irreversible sealing of carrier micropores by polymeric components contained in the crude mixture (i.e., ferulic acid polymer) or other contaminants, whose amount increases when a more concentrated laccase mixture is used. On the basis of the abovementioned results, the optimal parameters selected are (i) solids activation with 0.5% glutaraldehyde; (ii) 1 h incubation with laccase mixture (50 mM sodium phosphate buffer, pH 6.5, and 80 IU/g support) at room temperature. In these conditions, a maximum immobilization yield of about 70% was achieved.

As expected, there is a difference between the actual laccase activity expressed by the biocatalyst and the activity loss in the liquid supernatant. This effect may be due to the modifications of the enzyme structures occurring during covalent immobilization.

**Figure 3:**
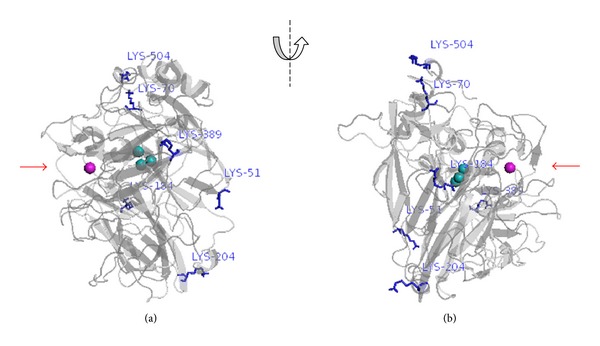
Three-dimensional model of POXC, elaborated with Pymol [27], with accessible lysine residues shown as stick. The protein is showed in two opposite orientations, wherein the access to the active site is indicated by a red arrow. T1 copper is shown as a magenta sphere. Copper atoms of the trinuclear cluster are displayed as cyano spheres.

The analysis of POXC primary sequence coupled to the examination of protein 3D model reveals the presence of six Lys residues, all of them localized on the protein surface and potentially available for the covalent bonding with glutaraldehyde. As shown in Figure 3, all Lys residues are mapped on the opposite side with respect to the active site; thus enzyme binding in nonproductive orientation is less probable. On the other hand, multipoint attachment of protein to support seems to be favored since two couples of lysine residues very close to each other are identifiable on the protein surface (Lys 504 and Lys 70 in Figure 3(a); Lys 51 and Lys 20 in Figure 3(b)). Such kind of interaction with the support may be responsible for distortion of enzymatic conformation, causing a decrease in its activity [10].

### 3.2. Assessment of Immobilized Biosystem Performances

The performances of the immobilized biosystem have been tested by assessing its stability and catalytic parameters.

#### 3.2.1. Stability Parameters

**Figure 4:**
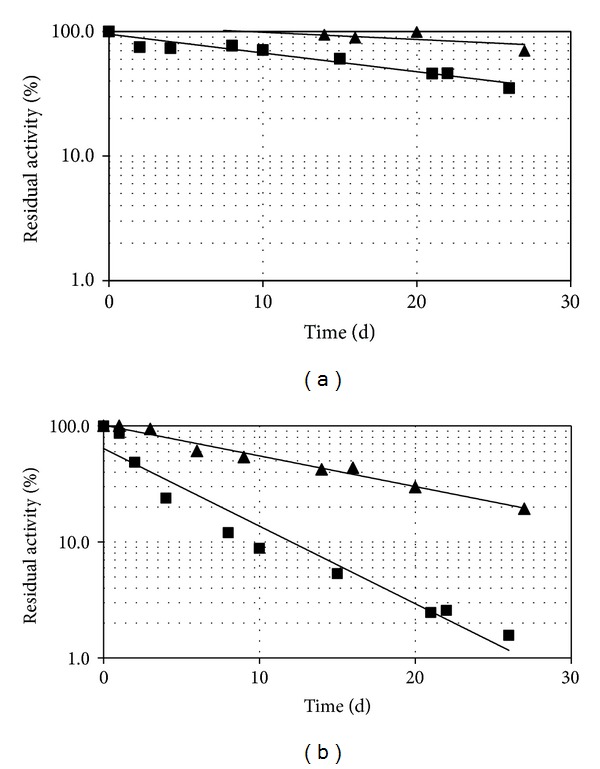
Stability of immobilized and free laccase mixture monitored at 4°C (a) and at room temperature (b). Black squares and free enzyme; black triangles and immobilized enzyme.

In order to have a suitable amount of immobilized activity on an easily handling amount of solid support, an enzyme to support ratio of about 290 IU/g was used, obtaining, with the aforesaid optimized conditions, an immobilization yield of about 45%. Storage stability of the immobilized laccase mixture has been monitored both at room temperature and at 4°C and was compared with that observed for free laccase mixture. Residual activity was calculated and expressed as percentages of residual activity at different time intervals. As showed in Figure 4, the immobilized mixture, stored at room temperature, displays about 7-fold increased stability with respect to the free laccase mixture (*t*_1/2_ free enzyme = 1.6 days; *t*_1/2_ immobilized enzyme = 11.6 days). On the contrary, stability at 4°C shows a 3-fold increase of *t*_1/2_ (*t*_1/2_ free enzyme = 18.5 days; *t*_1/2_ immobilized enzyme = 61 days).

The enhanced stability of immobilized laccase may be due to the prevention of structural rearrangement and the lower flexibility of the immobilized form, both caused by multipoint attachment to the support [28]. These results are consistent with those described in other reports of covalent laccase immobilization on silica-based supports such as kaolinite or mesoporous silica nanoparticles [15, 29–32]. In most cases in which glutaraldehyde was used as crosslinking agent, immobilization process exhibits low laccase recovery but improvements in the operational stability and stability against denaturing agents are evident [16]. Liu et al, for example, reported an improvement of both thermal and operational stability of laccase, when silanized and glutaraldehyde activated silica nanoparticles were used as supports, as illustrated by the retention of 61% of the residual activity after 4 h at 60°C and the retention of 55% of the activity after 10 cycles of operation.

Immobilization can provide an artificial microenvironment surrounding the enzyme that can alter surface-exposed hydrophilic and/or charged groups and their electrostatic interactions and thus influencing protein structure and function. In particular, in the immobilized system described in this study, enzymes could be doubly protected by thermal inactivation thanks to the higher number of positive charges on the surface and to the increased hydrophobicity, both provided by silanization [33].

#### 3.2.2. Catalytic Parameters

In order to perform a kinetic characterization of the immobilized biocatalyst, purified laccase POXC from* P. ostreatus* has been used rather than the crude mixture. The purified enzyme has been immobilized in the following conditions: 0.2 g of activated perlite was incubated for 1 h with purified POXC solution (50 mM NaP buffer, pH 6.5, and 250 IU/g of support as initial activity) at room temperature, resulting in about 40% immobilization yield (100 IU/g support). Kinetics of immobilized POXC against ABTS has been assessed by means of the circulating fixed bed reactor commonly employed for activity measurements. Results provided *K*_*M*_ = 0.44 mM and *K*_cat_ = 1.2 · 10^4^ min^−1^. Comparing these parameters with those characteristics of POXC in liquid phase (*K*_*M*_ = 0.03 mM, *K*_cat_ = 6.2 · 10^5^ min^−1^), both a decreased value for *K*_cat_ and a higher value for *K*_*M*_ were found. These differences could be a consequence of either the loss of conformational integrity of the immobilized enzyme due to multipoint attachment, as also reported for different immobilized biocatalyst [10], or of lower accessibility of substrate to the active sites of the immobilized enzyme, caused by enzyme overcrowding on support surface.

### 3.3. Dye Adsorption

The immobilized enzyme mixture was tested for its decolourisation potential against the model anthraquinonic dye Remazol Brilliant Blue R (RBBR). A first series of tests was addressed to assess the extension of dye adsorption on solids surface. This characterization was carried out to discriminate between dye conversion by immobilized enzyme and dye adsorption: the enzymatic conversion could be masked by a rapid adsorption kinetics and/or large adsorption capacity of the solid support. Moreover, the adsorption of dyes as well as adsorption of their oxidized products on the immobilization support could inactivate/inhibit the enzyme [34]. The assessment of the RBBR on the activated carrier could allow adopting strategies to minimize any adverse effect on dye conversion.

Tests were carried out with both untreated particles and activated particles. No adsorption of RBBR was observed on the inert particles. Tests carried out with samples prepared adopting the optimal conditions defined in the previous section reported an adsorption of about 71 mg_RBBR_/g_perlite_. Therefore, the adsorption phenomenon is related to the functional groups from silanization or glutaraldehyde activation process. Enzymatic assays performed after dye saturation showed that immobilized laccase activity was completely inhibited.

The reported findings inspired modifications of the immobilization protocol to limit dye adsorption to a negligible value. The effect of several parameters on adsorption capacity and immobilization yield was investigated. In particular, the parameters affecting the surface properties of the support (i.e., APTS concentration and pH, temperature, and concentration for glutaraldehyde crosslinking) were tuned. Results are summarized in Table 2. The adsorption capacity of the silanized perlite rises up to 96.1 mg_RBBR_/g_perlite_ (A2 in Table 2). As a matter of fact, by reducing the APTS concentration from 4 to 0.4% volume, keeping the other conditions constant, the adsorption capacity was approximately halved and an undesired drop of immobilization yield was observed. The extent of perlite silanization determined the concentration of active amino groups on the carrier surface; this parameter likely plays a key role in the adsorption of the dye. Thus, the improvement of the condition for glutaraldehyde activation would provide a reliable solution to saturate the free amino groups available for the interaction with the anionic dye. In run A4, two consecutive activation steps with glutaraldehyde were assessed. However, adsorption was not significantly affected by this treatment. Rising of the pH from 6.5 up to 8 during glutaraldehyde reaction provided a further decrease of the immobilization yield down to 16.4%, together with a negligible effect on adsorption capacity (compare A5 with A3). This effect was assessed on carrier silanized with 4 or 0.4% APTS solutions (A5-A6).

An effective reduction of RBBR loading (32.9 mg/g), coupled with a satisfying immobilization yield (51.2%), was achieved by incubating the solid silanized with 0.4% APTS with 1% glutaraldehyde at pH 8 and 60°C [25]. The latter operating conditions were selected for further investigation of dye degradation by laccase immobilized on perlite.

### 3.4. Decolourisation Experiments

Preliminary tests carried out in fixed beds filled with of enzyme-immobilized particles pointed out that this reactor system does not fit with the features of the coated particles. As a matter of fact, the bed made of inert particles was successfully operated even though a high pressure drop was measured. However, clogging phenomena were recorded when particles with immobilized enzymes were adopted: liquid stream flowed very slowly notwithstanding the high pressure drop adopted across the bed.

The fluidized bed was chosen to get round the fixed bed constraint [35, 36]. Liquid flow rate required for particles fluidization was supplied by the recirculating stream while the stream containing the dye was continuously supplied at the flow rate related to the residence time suitable for dye conversion.

**Figure 5:**
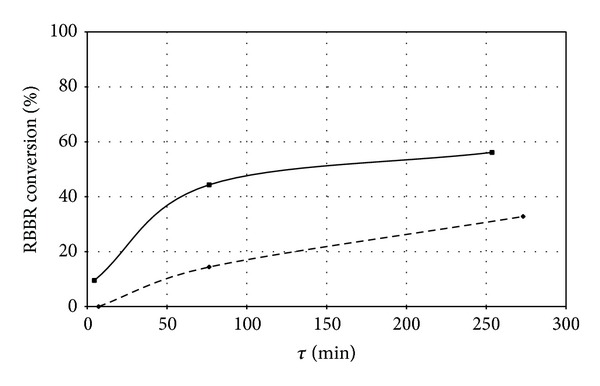
RBBR conversion degrees versus reactor space-time (*τ*). Data refer to decolourisation experiments carried out in fluidized bed reactor operated with immobilized laccases: sample C1 (dashed line); sample C2 (solid line).

Preliminary decolourisation experiments were carried out adopting two batches of immobilized enzyme: samples C1 and C2 reported in Table 3 with the operating conditions adopted for the immobilization and samples C1 and C2 area characterized by comparable dye adsorption capacities and different amount of immobilized laccase activity. The initial activity of sample C1 was 10 IU/mL and that of sample C2 was 28 IU/mL.  Table 4 reports the dye conversion degrees measured under steady state conditions as a function of the reactor space-time *τ*. Dye conversion degrees versus the reactor space-time are plotted in Figure 5.

The maximum conversion degree was measured for tests carried out with sample C1: 33% at *τ* = 4.6 h. Performance measured with the C2 sample were definitively satisfactory: 56.1% conversion at *τ* = 4.2 h. Decolourisation runs with both samples C1 and C2 lasted about 80 and 160 h with a total volume of treated RBBR solution of 3.5 and 4.5 L, respectively. Comparing the results of the decolourisation tests carried out with samples C1 and C2, it could be inferred that the better performance achieved by sample C2 was due to its higher activity per volume unit of carrier. It is remarkable to note that (i) in both decolourisation experiments residual enzyme activity was about 30% of the initial activity; (ii) both samples are characterized by almost the same dye adsorption capacity (about 30 mg/g carrier). These results suggest that the biocatalyst deactivation is mainly related to adsorption phenomena occurring at the same extent for both samples. Twofold reason suggests ruling out the effect of the extent of conversion on biocatalyst deactivation within the operation time investigated (about 160 h): (a) the same deactivation extent has been measured even though different conversions have been measured; (b) constant conversion was observed under steady state conditions for long time.

The analysis of reported results suggests that the procedure adopted for the preparation of sample C2 (Table 3) produced a catalyst characterized by satisfactory values of immobilization yield, for example, satisfactory rate of decolourisation, and low adsorption capacity of RBBR. The latter feature provides the minimum deactivation extent related to the fast dye adsorption phenomenon that initially competes with the dye conversion during decolourisation tests.

## 4. Conclusions

In the present study, a crude laccase preparation from* P. ostreatus* was successfully immobilized on perlite, a cheap porous silica material. Optimization of process experimental parameters was performed. Stability and catalytic parameters of the immobilized laccases were compared with those of free enzyme. Remarkable results show that the stability increases as a consequence of enzyme immobilization but a reduction of the catalytic performances in terms of ABTS conversion kinetics was also observed. These results are in agreement with those expected phenomena occurring as consequences of enzyme immobilization on solid supports [37], for example, reduced catalytic activity and/or increased stability with respect to the soluble counterpart as results of electrostatic and partitioning effects in the immobilized enzyme microenvironment, of conformational losses inside the pores of the support, and external and internal mass transfer phenomena. Moreover, decreased protein flexibility resulting from multipoint attachment and/or enzyme overcrowding on the surface of the support [38] are also responsible for impaired catalytic performances of the immobilized system. Concerning the optimization of immobilization protocol, it can be concluded that the large achieved yield of immobilization, the enhanced stability of the immobilized biocatalyst together with the relatively low cost of both the crude enzyme extract, and the support encourage the exploitation of such biosystem in different industrial application demanding laccases.

Part of the study was devoted to the assessment of the performances of the immobilized laccase in terms of continuous conversion of a reference dye. Accordingly, the immobilized system was tested for RBBR conversion in a fluidized bed recycle reactor. Results obtained in this work showed that the observed RBBR decolourisation by immobilized laccase is mainly due to enzyme action despite the occurrence of dye adsorption-related enzyme inhibition. In this regard, fine tuning of immobilization conditions has allowed balancing the immobilization yield and the resulting rate of decolourisation, with the adsorption capacity of the solid biocatalyst. In the continuous lab scale reactor, a maximum conversion degree of 56.1% was achieved at reactor space-time of 4.2 h.

In conclusion, the reported data may inspire further work concerning the applicability of such immobilized biocatalyst to the treatment of wastewaters containing dyes. Kinetic parameters of dye conversion as well as of deactivation processes (adsorption-related and long-term deactivation) may be inferred, in order to provide tools for the rational design of continuous decolourisation processes with immobilized biocatalysts.

## Figures and Tables

**Table 1:** Effects of experimental parameters on laccase immobilization yield on perlite. NaC: 50 mM sodium citrate buffer; McI: McIlvaine's buffer. *Incubations were performed in 50 mM sodium phosphate buffer, unless otherwise is indicated. Reported data are the mean values of three independent experiments. The standard deviation of tests carried out at a given set of operating conditions was less than ±10%.

Run	Initial activity (IU/g)	Initial protein (mg/g)	Glutaraldehyde % vol	pH*	Incubation time and temperature	Immobilized activity (IU/g)	Immobilization yield (%)
R1	550	4.5	1	6.5	4 h RT	200	36
R2	550	4.5	1	6.5	Overnight 4°C	128	23
R3	550	4.5	2.5	6.5	4 h RT	206	37
R4	550	4.5	2.5	6.5	Overnight 4°C	151	27
R5	550	4.5	5	6.5	4 h RT	206	37
R6	550	4.5	5	6.5	Overnight 4°C	207	38
R7	550	7.75	1	6.5	1 h RT	160	29
R8	550	7.75	1	6.5	4 h RT	161	29
R9	550	7.75	0.5	6.5	1 h RT	186	34
R10	550	7.75	0.5	6.5	4 h RT	178	32

R11	80	1.3	0.5	6.5	1 h RT	56	70
R12	285	4.75	0.5	6.5	1 h RT	136	48
R13	800	11.25	0.5	6.5	1 h RT	199	25

R14	500	4.25	0.5	6.5	1 h RT	173	35
R15	500	12	0.5	6.5	1 h RT	173	35
R16	500	22.5	0.5	6.5	1 h RT	184	37

R17	275	2.25	0.5	NaC 5.5	1 h RT	52	19
R18	275	2.25	0.5	6.5	1 h RT	137	50
R19	275	2.25	0.5	7.5	1 h RT	120	43
R20	275	2.25	0.5	McI 5.5	1 h RT	32	12
R21	275	2.25	0.5	McI 7.5	1 h RT	66	24
R22	275	2.25	0.5	McI 6.5	1 h RT	42	15

**Table 2:** Adsorption capacity of treated perlite samples obtained in different experimental conditions. Data are average values of three independent experiments. The standard deviation of each series of results was less than ±10%.

Perlite sample	APTS (%)	Glutaraldehyde activation	Adsorption capacity (mg RBBR/g solid)	Immobilization yield (%)
A1	4	0.5% (pH 6.5)	71	45
A2	4	—	96	—
A3	0.4	0.5% (pH 6.5)	28	29
A4	4	0.5% (pH 6.5, 2X)	64	—
A5	0.4	0.5% (pH 8)	31	16
A6	4	0.5% (pH 8)	64	38
A7	0.4	1% (pH 8 60°C)	33	51

**Table 3:** Immobilized enzyme samples employed in decolourisation experiments. Initial and final activity refer to laccase activity measured on solid carrier at the beginning and at the end of the decolourisation experiment.

Perlite sample	Immobilization conditions	Adsorption capacity (mg_RBBR_/g_solid_)	Initial activity (IU/mL)	Immobilization yield (%)	Final activity (IU/mL)
C1	0.4% APTS, 0.5% Glut pH 6.5	27.5	10	23	3
C2	0.4% APTS, 1% Glut pH 8.5 60°C	33	28	51	9

**Table 4:** Decolourisation experiments with laccase immobilized on perlite.

Perlite sample	Inlet RBBR concentration (mg/L)	Recirculation flow rate, *Q_r_* (mL/min)	Total volume (mL)	Dye-feeding rate, *Q* (mL/min)	Recirculation ratio	*τ* (min)	Outlet RBBR concentration (mg/L)	Dye conversion (%)
C1	41.5	13	71	10 0.9 0.3	— 14 50	7.1 76 273	41.5 35.5 27.9	— 14 33

C2	39.1	13	71	16 0.9 0.28	— 14 46	4.4 76 253	35.4 21.8 17.2	9.5 44 56
